# Supplementary material for: Modelling Microglial Innate Immune Memory In Vitro: Understanding the Role of Aerobic Glycolysis in Innate Immune Memory
Source: Int J Mol Sci. 2023 May 18;24(10):8967. doi: 10.3390/ijms24108967 (PMC10219556; doi:10.3390/ijms24108967)
Supplement: Supplementary file 1 [file ijms-24-08967-s001.zip › Figures S1 and S2.pdf]

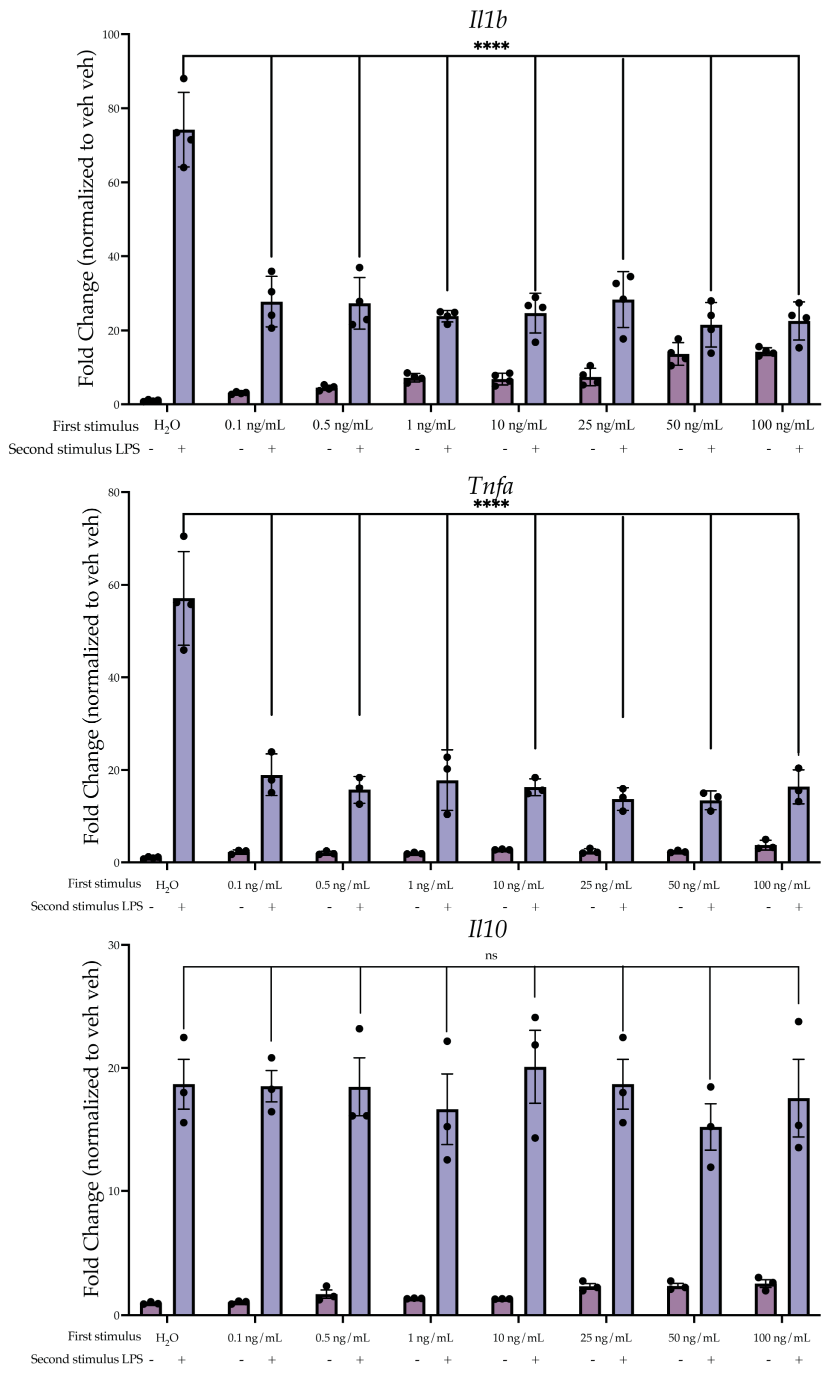

Figure S1: LPS induces tolerance regardless of dose *in vitro.* RT qPCR assessment of gene expression of *Il1b, Tnfa,* and *IL10* in BV2 cells treated with two stimuli of H_2O or LPS. Gene expression assessed 3 hours after the second hit. n=1 independent experiments, 3 replicates per experiment. qPCR shown as bar graph of Log_2(Fold Change) + SEM Tukey's post hoc significances denoted (**** p<0.0001).

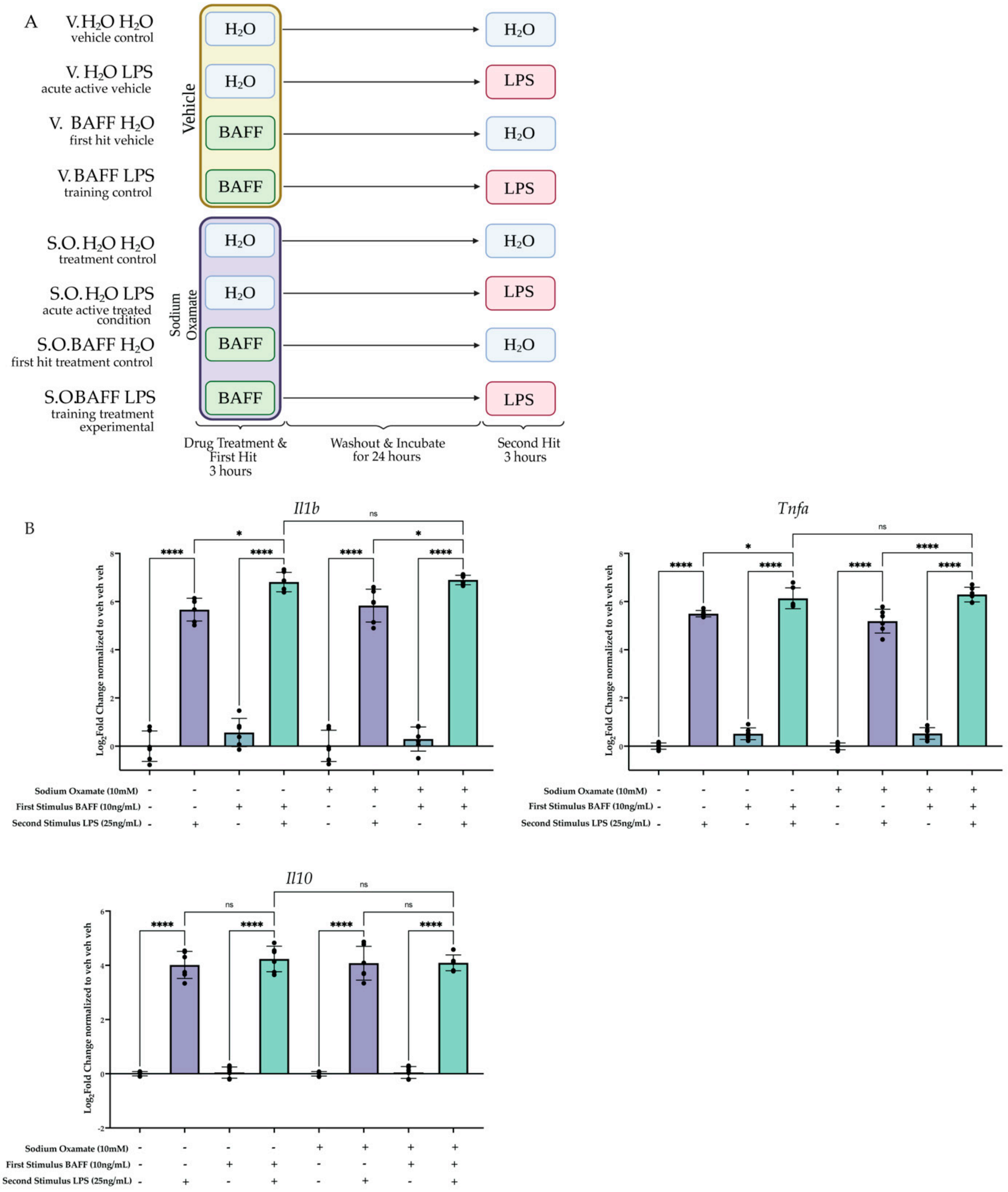
A
V.H2O H2O
vehicle control
V. H2O LPS
acute active vehicle
V. BAFF H2O
first hit vehicle
V. BAFF LPS
training control
S.O.H2O H2O
treatment control
S.O.H2O LPS
acute active treated condition
S.O.BAFF H2O
first hit treatment control
S.OBAFF LPS
training treatment experimental
Vehicle
Sodium Oxamate
H2O
BAFF
LPS
Drug Treatment & First Hit 3 hours
Washout & Incubate for 24 hours
Second Hit 3 hours
B
Il1b
Tnfa
Il10
Log2Fold Change normalized to veh veh
ns
Sodium Oxamate (10mM)
First Stimulus BAFF (10ng/mL)
Second Stimulus LPS (25ng/mL)

Figure S2: Sodium Oxamate treatment does not impact BAFF pretreatment induced training. (A) BV2 cells were treated with Sodium Oxamate (10mM) or vehicle concurrently with H_2O or BAFF (10ng/mL) for 3 hours followed by washout. After 24 hours of incubation, the cells were treated with H_2O or LPS (25ng/mL) for 3 hours prior to analysis. (B) RT-qPCR analysis of gene expression of proinflammatory cytokine *Il1b* and *Tnfa* assessed 3 hours after the second stimulus of LPS or H_2O. n=3 independent experiments, 2 replicates per experiment. (C) RT-qPCR analysis of gene expression of anti-inflammatory cytokine *Il10* assessed 3 hours after the second stimulus of LPS or H_2O. n=3 independent experiments, 2 replicates per experiment. qPCR shown as bar graph of Log_2(Fold Change) + SEM Tukey's post hoc significances denoted (*p<0.03, **** p<0.0001)
